# Factors influencing access to early intervention for families of children with developmental disabilities: A narrative review

**DOI:** 10.1111/jar.12852

**Published:** 2020-12-22

**Authors:** Suzi J. Sapiets, Vasiliki Totsika, Richard P. Hastings

**Affiliations:** ^1^ Centre for Educational Development, Appraisal and Research (CEDAR) University of Warwick Coventry UK; ^2^ Division of Psychiatry University College London London UK; ^3^ Department of Psychiatry, School of Clinical Sciences at Monash Health Monash University Clayton VIC 3800 Australia

**Keywords:** autism, developmental disabilities, disparities, early intervention, intellectual disabilities, service utilization

## Abstract

**Background:**

Early intervention (EI) can improve a range of outcomes for families of children with developmental disabilities. However, research indicates the level of access does not always match the level of need. To address disparities, it is essential to identify factors influencing access.

**Method:**

We propose a framework where access to EI is conceptualised as a *process* that includes three main phases. A narrative review examined potential barriers, facilitators and modifiers of access for each phase.

**Results:**

The process of access to EI includes the following: 1) recognition of need, 2) identification or diagnosis and 3) EI provision or receipt. Several factors affecting access to EI for each phase were identified, related to the family, services, the intersection between family and services, and the context.

**Conclusion:**

A broad range of factors appear to influence the process of access to EI for this population. Our framework can be used in future research investigating access. Broad implications for policy, practice and future research to improve access to EI are discussed.

## 1 INTRODUCTION

Access to early intervention (EI) has been identified as a priority for global research in developmental disability (Collins et al., 2017; Tomlinson et al., 2014). As a term, developmental disability refers to several developmental conditions but especially developmental delay, intellectual disability and autism spectrum disorder (ASD)^1^. The onset of developmental disabilities takes place during early development and lasts throughout an individual's lifetime (Patel & Merrick, 2011). Whilst the individual needs of children with developmental disabilities are unique, general delays in cognitive and adaptive skills are present by definition (American Psychiatric Association, 2013; Carulla et al., 2011; McDonald et al., 2006). A range of other adverse outcomes are associated with developmental disabilities, such as poorer mental health, poorer physical health and social inequalities (Emerson, 2003; Emerson & Hatton, 2007; Gurney et al., 2006; Vasilopoulou & Nisbet, 2016). For example, an increased risk for behaviour problems is present in children with developmental disabilities as young as 3–5 years old (Totsika et al., 2011), and this increased risk persists into adolescence and adulthood (Gray et al., 2012). Parental, especially maternal, well‐being is also poorer in families of children with developmental disabilities and this group difference also emerges early in the child's life (Hastings, 2016). The presence of such outcomes for children with developmental disabilities and their families means it is critical to consider the provision of EI.

EI is an umbrella term which encompasses a range of different supports to promote optimal child development, such as interventions targeted to improve child and/or family outcomes, and general contact with support services (e.g. education, health, social care services) in the early years (Akhmetzyanova, 2016; Brito & Lindsay, 2015; Dunst, 2007; Munro, 2011; Sharp & Filmer‐Sankey, 2010). In the present review, we conceptualise EI as formal support accessed by families of children with suspected or diagnosed developmental disabilities during early childhood (i.e. 0–6 years of age), including contact with various universal and specialist services (e.g. education, health, social care), in addition to specific intervention programmes (Brito & Lindsay, 2015). EI for developmental disabilities can include both preventative programmes, such as universal or targeted developmental screening, or responsive support, such as service provision following diagnosis of developmental disability or identification of a child or family need (e.g. child sleep problem, parental mental health) (Munro, 2011). Child developmental outcomes are influenced by various systems in the child's environment and interactions within and between systems (Bronfenbrenner, 1979; Dunst, & Trivette, 2009; Guralnick, 2001). For example, in addition to individual child characteristics, ecological system theory indicates child development is influenced by five environmental systems: (a) microsystems, the child's direct environment for interaction (e.g. with family, friends, professionals supporting them), (b) mesosystems, interactions between two microsystems (e.g. interactions between parents and professionals), (c) exosystems, indirect environments that influence child development (e.g. a parent's workplace, local government), (d) macrosystems, attitudes and beliefs within culture and society and (e) chronosystems, interactions between the various systems and their influence on each other over time (Bronfenbrenner, 1979; Rose & Tudge, 2013). As the provision of EI solely to the child may not be as effective (or unsustainable) at improving child development, our definition of EI includes support provided to the family system (cf. Akhmetzyanova, 2016), including the provision of support for the child, parental caregivers, siblings, other family members or a combination of these.

Early identification of developmental disabilities and access to EI at the earliest possible age can improve several outcomes, including child development and adaptive skills (Lai et al., 2014; Majnemer, 1998; Ryberg, 2015; Smith et al., 2015), behaviour problems and sleep (Roberts et al., 2003; Wiggs & Stores, 2001) and parental mental health and self‐efficacy (Bristol et al., 1993; Sofronoff & Farbotko, 2002). As a result, EI access has the potential to increase quality of life for families and their children with developmental disabilities. EI can have societal economic benefits by reducing the economic strain of costly services later in life, though further research is needed to quantify potential or actual cost savings (Knapp et al., 2009; Motiwala et al., 2006; Piccininni et al., 2017). Further, early identification and EI are advocated for in international policy, practice and guidance documents (e.g. Collins et al., 2017; Individuals with Disabilities Education Act (IDEA), 2004; National Disability Insurance Scheme (NDIS) Act, 2013; United Nations Convention on the Rights of the Child (CRC), 1989; United Nations Sustainable Development Goals (SDGs), 2015; World Health Organisation & Unicef, 2012).

Despite the strong case for EI, research indicates the level of access to EI does not always match the level of need (Betz et al., 2004; Crane et al., 2016; Gobrial, 2012; McManus et al., 2014; Stevens, 2006). For example, in a sample of 965 children with a range of developmental disabilities in the USA, McManus et al. (2014) found less than half (45.7%) accessed EI. Ruble et al. (2005) found the use of Medicaid services for children with ASD in the USA was only 10% of the numbers expected from ASD prevalence rates. Furthermore, in a study of over 1000 parents in the UK, during or following the ASD diagnostic process, only 21% were directly offered support, 38% were signposted to advice or help, and 35% were not offered any help or assistance at all (Crane et al., 2016).

The timeliness of EI access is also an issue, as earlier intervention can significantly alter the developmental trajectory of children with (or at‐risk of) developmental disabilities (see Webb et al., 2014). However, low rates of access to EI have been found for very young children (i.e. <3 years of age) with developmental disabilities (Grant & Isakson, 2013; McManus et al., 2014; Roberts et al., 2007; Rosenberg et al., 2008). For example, Rosenberg et al. (2008) found only 10% of children with developmental delays received EI at age two, despite being eligible under Part C of IDEA, a federal law that mandates EI services in the USA. Notably, lower rates were found in Grant and Isakson (2013). Across the USA, only 2.7% of age‐eligible children (birth through 35 months of age) received EI under Part C of IDEA, with a range of 1.2–6.5% for specific states, suggesting there is a significant group of children who need but do not receive EI (Grant & Isakson, 2013). Delays in diagnosis receipt may also be long, especially for ASD (Crane et al., 2016; Howlin & Moore, 1997; Wiggins et al., 2006), which may further delay or prevent access to EI, especially where a diagnosis is needed to access services.

Families may also experience difficulties in the identification of (and access to EI for) other child and family needs. For example, whilst 88 of 102 parents of children with developmental disabilities in the USA studied by Betz et al. (2004) reported child behaviour concerns, only 12 were referred for services. A lack of support to meet the needs of parents and siblings of children with developmental disabilities has also been reported (Bromley et al., 2004; Burke & Montgomery, 2000). For example, in Bromley et al.’s (2004) study of 68 mothers of children with ASD in the UK, several unmet needs related to parental and other family support, such as ‘To do things parent enjoys’ (91%), ‘Break from caring for child’ (87%), ‘Someone to talk to’ (85%) and ‘To enable parent to spend more time with other children’ (63%). Inequities in access to EI have also been identified. For example, disproportionately low rates of access to developmental surveillance and diagnosis of developmental disabilities are found in families from ethnic minority groups in the USA and Australia (Mandell et al., 2009; Overs et al., 2017).

To improve outcomes for children with developmental disabilities and their families, it is essential to first develop a comprehensive understanding of factors related to EI access for this group. Theoretical frameworks aiming to describe differences in EI access have primarily focused on the family seeking or accepting EI (Arcia et al., 1993; Birkin et al., 2008). Arcia et al.’s (1993) model suggested families’ willingness to seek and enter EI is influenced by predisposing family factors, perception of the ‘problem’, and enabling factors (i.e. the intersection of family factors and EI). Birkin et al.’s (2008) model additionally included EI‐related factors, such as clinical relevance, cultural relevance and accessibility. Whilst both models are informative, EI access is a process that spans across various systems (the family system, the system of service provision, etc.) and requires a framework that can encompass this complexity. Therefore, the present paper aims to: a) propose a conceptual framework that maps the process of access to EI and b) use this framework to synthesise an overview of factors that might influence the process of access to EI for families who have children with developmental disabilities.

## 2 PATHWAY OF ACCESS TO EARLY INTERVENTION

**FIGURE 1:**
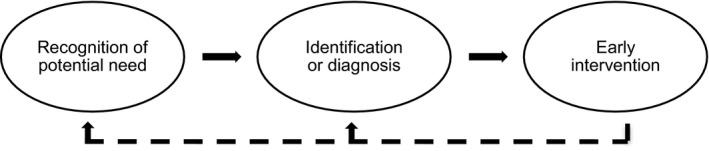
Pathway of access to early intervention

Access to EI is a *process*, as opposed to a time‐specific phenomenon. We propose that this process might be summarised by Figure 1, represented as a general pathway of access to EI. This framework shows the temporal sequence of steps required for EI access: recognition of potential need; identification or diagnosis; and, finally, receipt or provision of EI. Our proposed pathway is intentionally generic to enable the exploration of access to different services and supports in various contexts. It also enables the consideration of actions instigated by different systems, such as families, professionals, services or systems; each of which has an important role in the process. Furthermore, this process can happen at different time points in the lifecycle of a family and multiple times in a response to different needs or different EI provisions (i.e. child or family focused EI). The process may, therefore, be cyclical and potentially bidirectional, also indicated in Figure 1.

The first phase of the pathway is the recognition of potential need in the family system (referred to hereafter as ‘recognition’). The ‘need’ can relate to a number of different areas, such as a need to support the child (e.g. development, physical or mental health, educational, behavioural), parents and carers in their role supporting the child (e.g. educational, psychological, social, mental health), other family members (e.g. sibling support) or other family life domains (e.g. housing, monetary support, transport). Initial recognition can be made by a parent, other family member, someone in the family's network, a professional working with the family, or through universal monitoring and screening systems. For example, a study exploring parental experiences of intellectual disability and ASD diagnosis in Scotland reported 60% of parents raised initial concerns about their child's development, whilst 40% were made aware of the concern by others (Pankaj, 2015). This phase may relate to the recognition of other needs within the family system, such as a need for sibling support (Dyke et al., 2009). To progress through phases, the potential need recognised will need to be shared with another party (a parent, professional or service). If recognition of potential need is not shared, or the need is resolved, the process might stop at this phase.

The second phase covers formal identification or diagnosis of need (referred to hereafter as ‘identification’), which may involve a referral for screening or assessment of the need, the screening or assessment itself, and the formal identification or diagnosis of need and associated supports. Whilst this predominantly relates to the identification of developmental disabilities, it also encapsulates the identification of other family needs, such as the identification of parental needs via a carer's assessment (McCafferty and McCutcheon, 2020). If a need is not formally identified, the process may stop at this phase, or monitoring may be put in place (Guralnick, 2001). Once a need is formally identified, the next steps are planning appropriate support and putting it in place.

The final phase is the provision or receipt of EI, which may include the provision of information and advice, signposting to services or receipt of support from a range of services and interventions. The support provided will vary depending on the need and the associated impact on the child and family. Accessing support may not be the end of the process; families may return to an earlier point in the pathway for a number of reasons, such as requiring further support to meet the need, a change in the family's situation, or to access support for a different need within the family system.

Various factors might influence the process of access to EI, and the way these factors operate may vary for the three different phases. Using our framework depicting the pathway of access to EI might be a useful starting point to identify factors that influence access to EI for families of children with developmental disabilities, to develop understanding of why some families are not accessing support.

## 3 FACTORS INFLUENCING ACCESS TO EARLY INTERVENTION

To gain a comprehensive picture of potential factors associated with access to EI for developmental disabilities, we conducted a narrative review (see Ferrari, 2015). We considered evidence across broad fields (e.g. diagnostic services, paediatrics, health care, education, social care, family support, community services) and various developmental conditions within developmental disabilities (e.g. developmental delay, intellectual disability, ASD), rather than focusing solely on a specific EI or only one developmental disability category, such as ASD.

To ensure a minimum level of quality of the evidence, we only considered evidence from peer‐reviewed papers. To ensure a good match to our research question, we considered studies whose definition of EI matched ours (i.e. any formal support provided to families in early childhood, 0–6 years of age). In this broad field of investigation, factors associated with access to EI have been investigated in quantitative and qualitative studies with families, children and/or professionals. The majority of research evidence was from the USA or UK, with the rest from various other countries, such as Australia, Canada, China, New Zealand, Taiwan, India, Bangladesh, Singapore, Turkey and several European countries.

Below, we organise the description of factors according to the phases of the pathway of access to EI (recognition, identification and intervention; Figure 1) and their effect (i.e. barrier, facilitator or modifier). Factors were defined as barriers if they had a detrimental effect on a process (Hicks et al., 2007), such as factors that prevented, challenged or stopped something within the pathway of access. Facilitators were factors that had an enabling effect (Hicks et al., 2007), such as factors that sustained, enhanced, supported or allowed movement across the pathway. Lastly, modifiers were factors related to the process but did not have a direct impact on it, including factors that altered the relationship between another factor and its effect on the pathway; for example, parental education might affect parental awareness of services and language proficiency which in turn affect access (Bailey et al., 1999; Vande Wydeven et al., 2012).

**FIGURE 2:**
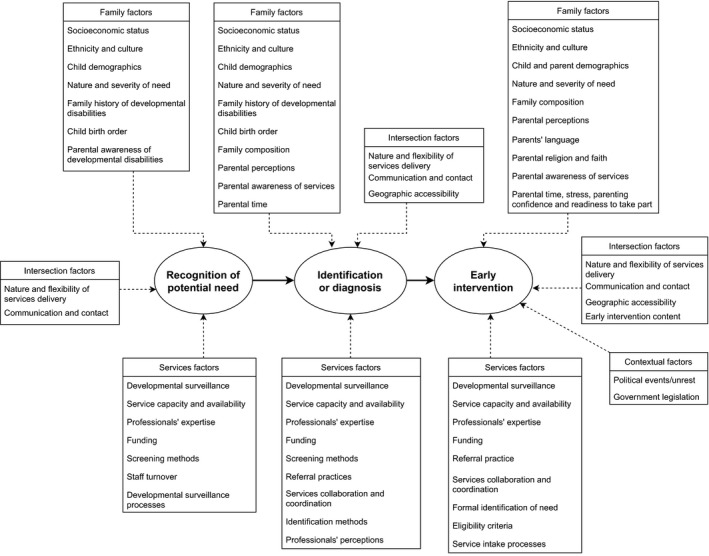
Factors influencing the pathway of access to early intervention

A variety of factors influencing the pathway of access to EI were identified through our review (see Figure 2 for an overview). Several factors operated at all three or two phases of the pathway of access to EI, whereas other factors appeared unique to one part of the process. An overview of the phases at which each factor operated is presented in Table 1 (see online supplement for full references).

**TABLE 1:** Factors influencing the phases of the pathway of access to early intervention

Factors	Phases influenced		
	Recognition	Identification	Early intervention
Family factors			
Parental socioeconomic status	✓	✓	✓
Ethnicity and culture	✓	✓	✓
Child age and gender	✓	✓	✓
Nature and severity of need	✓	✓	✓
Family history of developmental disabilities	✓	✓	
Child birth order	✓	✓	
Parental recognition and perceptions of need or EI		✓	✓
Parental awareness of services		✓	✓
Family composition		✓	✓
Parental time		✓	✓
Parental awareness of developmental disabilities	✓		
Parenting confidence			✓
Parental readiness to take part in EI			✓
Parents’ language			✓
Parental gender			✓
Parental religion and faith			✓
Parental stress			✓
Services factors			
Developmental surveillance	✓	✓	✓
Services capacity and availability	✓	✓	✓
Funding	✓	✓	✓
Professionals’ expertise	✓	✓	✓
Screening methods and tools	✓	✓	
Services collaboration and coordination		✓	✓
Referral practices		✓	✓
Staff turnover	✓		
Developmental surveillance processes	✓		
Identification methods and processes		✓	
Professionals’ recognition and perceptions of need		✓	
Formal identification of need			✓
Eligibility criteria			✓
Service intake processes			✓
Intersection factors			
Nature and flexibility of services delivery	✓	✓	✓
Communication and contact	✓	✓	✓
Geographical accessibility		✓	✓
EI content			✓
Contextual factors			
Political events/unrest			✓
Government legislation			✓

Note.

The phases of the pathway of access to early intervention are as follows: (a) recognition of potential need, (b) identification or diagnosis and (c) early intervention (Figure 1).

### 3.1 Recognition

#### 3.1.1 Family

Family factors (i.e. factors related to the family or child) that influenced the first phase, recognition, were parental socioeconomic status (SES), including parental economic status and educational level, parental awareness of developmental disabilities, ethnicity and culture, family history of developmental disabilities, child birth order, the nature and severity of the need, child age and child gender.

Higher parental SES facilitated recognition and lower SES was a barrier. For example, in Moh and Magiati’s (2012) study of ASD diagnosis in Singapore, parents with higher educational qualifications and income recognised potential child development problems earlier than parents with lower qualifications and income. The impact of parental SES on recognition might be magnified in contexts without a universally free healthcare system, in cases where the need is recognised by a professional. For example, in the USA an associated decrease in access to well‐child visits has been found for each month a child is without health insurance (Leininger & Levy, 2015), potentially obstructing recognition.

Being part of an ethnic minority group was a barrier to the recognition of developmental disabilities. For example, parents of children from ethnic minority groups in the USA reported a later age of child development concerns (Rosenberg et al., 2011). Culture also modified factors directly related to recognition, such as parental awareness of developmental disabilities. For example, low parental awareness of ASD was a barrier to recognition, which appeared more prominent in non‐Western cultures, such as Pasifika and Maori families in New Zealand (Birkin et al., 2008) and Somali families in the UK (Hussein et al., 2019).

Having a family member with developmental disabilities facilitated recognition. For example, Matheis and Matson (2015) found parents in the USA were more than twice as likely to accept routine ASD screening if their child had a family member with ASD (suspected or diagnosed). Another study in the USA found later birth order (i.e. child was second‐born or later, as opposed to first‐born) also facilitated recognition of ASD, whereas being first‐born was a barrier (Bickel et al., 2015). Rosenberg et al. (2011) demonstrated that a later age of child development concern was associated with first‐born children.

The nature (i.e. type) and severity of need influenced recognition. For example, a younger age of parental concern was found in parents of children with ASD compared to intellectual and other developmental disabilities in the USA (Zuckerman et al., 2015). Increased severity or increased number of needs also facilitated recognition. For example, a younger age of first parental concern was found in parents of children with co‐occurring ASD and intellectual disabilities, compared to parents of children with intellectual disabilities or ASD only (Zuckerman et al., 2015). In contrast, Matheis and Matson (2015) found where one developmental disability has already been identified, such as Down syndrome or cerebral palsy, parents may be less inclined to accept screening for another developmental disability, such as ASD. Findings from the same study also suggest younger child age and female gender were barriers to recognition, as parents of younger children and female children were more likely to refuse routine ASD screening (Matheis & Matson, 2015).

#### 3.1.2 Services

Services factors (i.e. factors related to professionals, services or governing systems) that influenced recognition were the implementation of developmental surveillance, including methods, tools and processes for developmental surveillance, professionals’ expertise, service capacity, staff turnover and funding.

The implementation of developmental surveillance was a facilitator, modified by the nature of the need and professionals’ experience (Dosreis et al., 2006; King et al., 2010; Nygren et al., 2012). For example, compared to other developmental disabilities, rates of routine screening were significantly lower for ASD, related to professionals’ expertise and lack of familiarity with ASD screening tools (Dosreis et al., 2006). Limited capacity of services, increased staff turnover, especially losing staff in managerial positions, and a lack of clarity around financial reimbursement were barriers to recognition of developmental disabilities through developmental surveillance (Dosreis et al., 2006; King et al., 2010; Roux et al., 2012).

The methods and tools utilised for routine developmental surveillance were not always effective at detecting potential developmental disabilities, which was a barrier to professionals’ recognition (King et al., 2010; Marshall et al., 2015). For example, several parents in Marshall et al. (2015) reported receiving a false‐negative prenatal screen for Down syndrome, delaying recognition. Utilising non‐traditional surveillance methods to contact a wider population (e.g. conducting telephone screening) facilitated recognition of developmental delay and ASD (Roux et al., 2012). Furthermore, utilising system‐wide processes for developmental surveillance, dividing staff responsibilities at multiple levels, and adjusting implementation systems based on active monitoring of implementation, facilitated recognition (King et al., 2010; Nygren et al., 2012).

#### 3.1.3 Intersection

Factors at the intersection of family and services that influenced recognition of need were the nature and flexibility of services delivery in relation to family factors, and communication between services and families. A good match between services delivery and family factors, and the ability to be flexible in services delivery, facilitated recognition. For example, Roux et al. (2012) facilitated recognition of developmental delay and ASD for families with low SES from ethnic minority groups by implementing developmental surveillance remotely. Employing professionals fluent in an array of languages also facilitated recognition, by reducing communication barriers (Roux et al., 2012).

### 3.2 Identification

#### 3.2.1 Family

Family factors that influenced the second phase, identification, were parental SES, ethnicity and culture, parental recognition and perception of need, parental knowledge of services, parental time resources, family history of developmental disabilities, family composition, child birth order, the nature and severity of need, child age and child gender.

Similar to the recognition phase, higher parental SES was generally a facilitator and lower SES was a barrier to identification (Fountain et al., 2011; Jimenez et al., 2014; Thomas et al., 2012). The financial set‐up of service systems modified the relationship between SES and identification. In contexts without a universally free service system, such as the USA, SES barriers reduced or disappeared when costs were removed or heavily subsidised (Jimenez et al., 2014), whereas low SES appeared to facilitate recognition in contexts with a universally free service system, such as the UK. For example, Brett et al. (2016) found an association between higher family deprivation and earlier diagnosis of ASD in the UK, indicating universal services lessen the impact of SES on identification. Despite the universal service system in the UK, some parents report having to pay privately for ASD diagnosis (Howlin & Moore, 1997), and a shorter ASD diagnostic period was experienced by parents’ who were able to pay for private services compared to parents’ dependant on public services (Keenan et al., 2010). Moh and Magiati (2012) found no association between income and ASD diagnosis in Singapore, which has a mixed service system. As services in Singapore are part funded by families’ mandatory savings and government subsidies, out‐of‐pocket costs are reduced, which may reduce economic barriers.

Being part of an ethnic minority group was a barrier to the identification of developmental disabilities. For example, Rosenberg et al. (2011) found being part of an ethnic minority group was a risk factor for delayed ASD diagnosis in the USA, such as multiracial or Black/African American ethnicity. Whilst SES accounted for some ethnicity disparities in the identification of developmental disabilities, due to an over‐representation of families from ethnic minority groups in low‐income communities (Thomas et al., 2012), Dababnah et al. (2018) found being part of an ethnic minority group persisted as a barrier regardless of SES for Black/African American parents of children with ASD in the USA.

Parental recognition of potential child need (e.g. developmental delay, ASD) facilitated identification, whereas parents’ non‐recognition or ambivalence of child need was a barrier (Bickel et al., 2015; Jimenez et al., 2012). Certain parental beliefs about the aetiology of developmental disabilities were barriers to identification, such as that developmental disability is caused by parenting style or is a punishment for the past behaviour of the family (Birkin et al., 2008). Parental beliefs about the causes of developmental disabilities were partly modified by religion and culture. For example, varied beliefs about the causes of ASD are documented across cultures, such as Maori, Pasifika, Korean, Somali and Taiwanese families (Birkin et al., 2008; Hussein et al., 2019; Shyu et al., 2010). Limited parental awareness of services, systems and processes was a barrier to the identification of developmental delay (Jimenez et al., 2014). Parental time constraint was also a barrier to the identification of developmental delay (Jimenez et al., 2012).

Having a family member with developmental disabilities facilitated identification. For example, having a sibling with ASD predicted an earlier age of ASD diagnosis for following children (Bickel et al., 2015). Fewer children living in the household facilitated identification, as it predicted earlier receipt of ASD diagnosis (Bickel et al., 2015). Furthermore, a later birth order (i.e. child was second‐born or greater, as opposed to first‐born) also facilitated identification of ASD, whereas being first‐born was a barrier (Bickel et al., 2015).

The nature and severity of need inevitably influenced identification. For example, in Crane et al.’s (2016) UK study, children with Asperger syndrome experienced longer diagnostic delays and later age of diagnosis compared to children with other ASD diagnostic labels (autism, Pervasive Developmental Disorder Not Otherwise Specified, Rett syndrome, autistic traits). Furthermore, children with special health needs, or delays in communication compared to other developmental domains, were more likely to be referred for development assessment in the USA, thus facilitating the identification of developmental disabilities (Jimenez et al., 2014; King et al., 2010). Generally, greater severity of need was a facilitator and lower severity was a barrier to being referred for developmental assessment in Canada (Shevell et al., 2001). An increased number of needs appeared to facilitate identification. In Jimenez et al.’s research (2014), for example, children were more likely to receive a developmental assessment if concerns covered more than one developmental domain. Furthermore, the identification of ASD was facilitated by the presence of co‐occurring intellectual disability (Rosenberg et al., 2011; Zuckerman et al., 2015). In contrast, Howlin et al. (1995) raised concerns regarding the diagnosis of ASD in children with Down syndrome due to diagnostic shadowing, whereby ASD symptoms are attributed to cognitive delays related to Down syndrome, obstructing the diagnosis of ASD.

Older child age appeared to be a barrier to identification. For example, compared to younger children, children aged >24 months were less likely to be referred to and receive a developmental assessment (Jimenez et al., 2014). However, after controlling for other variables, the only family factors associated with developmental referral or assessment were the nature and severity of need and child gender (Jimenez et al., 2014). Female child gender was a barrier to identification, especially the identification of ASD, whereas male gender was a facilitator (Begeer et al., 2013; Jimenez et al., 2014). Gender differences in autism diagnosis may also be linked to the nature of the need. For example, in Begeer et al. (2013), Asperger syndrome was the only ASD diagnostic label for which females were identified later than males. Differential presentation of ASD between females and males can mask diagnostic features of ASD in females, such as non‐verbal communication (Rynkiewicz et al., 2016), obstructing identification.

#### 3.2.2 Services

Services factors that influenced identification were the implementation of developmental surveillance and screening, referral practices, professionals’ recognition and perception of need, identification (i.e. assessment and diagnostic) methods and processes, professionals’ expertise, services capacity, availability, funding and collaboration.

Similar to recognition, developmental surveillance facilitated the number of children subsequently identified with developmental disabilities (King et al., 2010; Nygren et al., 2012). Furthermore, the methods and tools utilised for non‐routine developmental screening impacted identification as either a barrier or facilitator (King et al., 2010; Marshall et al., 2015; Roux et al., 2012). For example, Sices et al. (2009) found significant discordance between the outcomes of two commonly used developmental screening tools. The tools did not detect potential delays in the same children, with 33% of children being identified with a likely developmental delay through only one instrument.

Professionals’ proactive response to parental concerns and sending referrals directly to assessment services facilitated identification, whereas passive or reassuring responses to parental concerns, placing responsibility on parents to contact services and complex referral systems were barriers to the identification of developmental delay or ASD (Jimenez et al., 2014; Zuckerman et al., 2015). Referral practice was modified by the type of need, professionals’ recognition or perception of need, parental concerns or desire for referral, and screening tools utilised (Jimenez et al., 2014; King et al., 2010; Zuckerman et al., 2015). For example, in Zuckerman et al. (2015), passive or reassuring responses to parental concerns were higher amongst parents of children with ASD compared to other developmental disabilities, which was associated with longer diagnostic delays. Furthermore, professionals reported deferring or foregoing an assessment referral if they thought parents misunderstood screening questions (Jimenez et al., 2014).

Professionals’ recognition of need facilitated identification (e.g. conducted assessment, referred to another professional), whereas non‐recognition of need was a barrier (e.g. told parent there was no problem, reassured parent) (Crane et al., 2016; Zuckerman et al., 2015). Professionals’ perception of child need was modified by their occupational role, the nature and severity of need, child age, ethnicity and SES. For example, professionals’ perception of developmental disabilities in an artificial vignette varied dependent on their occupational role: psychiatrists more frequently identified ASD, and speech and language practitioners more frequently identified language disorder (Cuccaro et al., 1996). A study in the USA indicated a higher level of specialisation amongst professionals (measured by occupational role, for example specialist neurologists and psychiatrists) facilitated identification of ASD, whereas less expertise (e.g. primary care physicians) was a barrier (Kalkbrenner et al., 2011).

Assessment and diagnostic processes that were long, complex, and placed the onus on parents to obtain referrals and contact services, were barriers to identification of ASD in the UK (Howlin & Moore, 1997) and developmental delay in the USA (Jimenez et al., 2014). The use of standardised diagnostic tools (e.g. instruments, manuals) facilitated the identification of ASD. However, professionals did not always use diagnostic tools, reporting a preference for professional judgement, as tools were experienced as complex, time‐consuming and not always effective (Karim et al., 2012; Moh & Magiati, 2012; Wiggins et al., 2006). Utilising a multidisciplinary approach was reported as helpful to identify ASD but increased the assessment duration and occasionally led to conflicts of opinion (Moh & Magiati, 2012). Professionals’ perception of parental response to diagnosis also modified identification. Karim et al. (2012) found that professionals in the UK reported diagnosing children with Asperger syndrome, over another ASD diagnostic label, if they thought parents would respond better to this label.

Limited capacity and availability of services and professionals were also barriers to identification, including extensive waiting lists for ASD diagnostic assessment and a lack of specialists (Karim et al., 2012). Limited funding and resources within the system were barriers to identification, modified by budget allocation. For example, Karim et al. (2012) found UK government funding cuts to services increased barriers to ASD diagnosis. The development of a robust funding and business model, to sustain service provision through ongoing acquisition of local and federal grants, facilitated ASD identification in the USA (Mathews et al., 2018). Poor communication and collaboration between services and professionals were a barrier to the identification of developmental delay and ASD (Jimenez et al., 2012; Mathews et al., 2018).

#### 3.2.3 Intersection

Intersection factors that influenced identification were the nature and flexibility of services delivery in relation to family factors, communication between services and families, and geographical accessibility (i.e. the geographical intersection of services and families, such as geographical spread, proximity and urbanicity).

A good match between services delivery and family factors, and flexibility in services delivery, was a facilitator of identification. For example, delivering assessments in the family home rather than at a clinic, and providing support with transport, facilitated ASD identification amongst families with low SES in the USA (Carr & Lord, 2016).

Communication barriers that influenced identification included a loss of contact between services and families for various reasons (e.g. staff turnover, family moving), professionals not ‘listening’ to parents or addressing their perspectives, divergence in perceptions regarding needs, a lack of involvement of parents as partners and linguistic barriers (Howlin & Moore, 1997; Jimenez et al., 2012; Roux et al., 2012).

A general lack of services where families lived was a barrier to identification (Howlin & Moore, 1997). Increased urbanicity (indexed with indicators including population density, education level, occupation, age and rate of physicians per population) was a facilitator of identification of developmental disabilities, whereas lower urbanicity was a barrier, modified by the nature of the need (Chen et al., 2008; Kalkbrenner et al., 2011; Rosenberg et al., 2011). For example, Kalkbrenner et al. (2011) found a younger age of ASD diagnosis in areas with a higher concentration of specialist neurologists and psychiatrists in the USA. Whilst Chen et al. (2008) found living in rural areas was a barrier to obtaining an ASD diagnosis, they found no urbanicity differences present in the diagnosis of other developmental disabilities in Taiwan.

### 3.3 EI Provision or receipt

#### 3.3.1 Family

Family factors that influenced the third phase, EI provision or receipt, were parental SES, ethnicity and culture, parental perceptions of need or services, parental knowledge of services, their time resources, confidence, readiness for engagement in intervention, language, gender, religion and stress, family composition, the nature and severity of need, child age and child gender.

Again similar to recognition and identification, higher parental SES (i.e. increased education and financial resources) was a facilitator and lower SES was a barrier of EI receipt, modified by the financial set‐up of services (Jimenez et al., 2014; Marshall et al., 2015; Payakachat et al., 2017). Being part of an ethnic minority group was also a barrier to EI receipt. For example, in their study of access to services under Part C of IDEA in the USA, Rosenberg et al. (2008) found children from White ethnicity groups were more than twice as likely as children from Black ethnicity groups to receive EI. Although a higher proportion of children with health insurance received EI, insurance was not significantly associated with EI receipt when other factors were accounted for (ethnicity, developmental delay), perhaps as Part C services were available regardless of insurance status (Rosenberg et al., 2008).

Parental perceptions of developmental disabilities, their child's needs, and EI services influenced EI receipt, party modified by culture (Birkin et al., 2008; Chauhan et al., 2017; Evans et al., 2016; Shyu et al., 2010). For example, parents’ attributions of the causes of ASD influenced EI decisions in a Taiwan study (Shyu et al., 2010). In a study exploring EI use for families of children with developmental delays in India, Chauhan et al. (2017) found parents who perceived their child as physically weak often sought massage and medical services rather than educational services, associated with cultural perceptions of child development. A minimal perceived benefit of special education in relation to cultural traditions and expectations was a barrier to formal EI receipt, for example ‘What's the use of spending money [on special education] now if she cannot be married in a good family’ (Chauhan et al., 2017, p. 54).

Limited parental awareness of services, systems and processes was a barrier to EI receipt, modified by contact with professionals and the provision of information (Birkin et al., 2008; Chadwick et al., 2002). For example, in a study of parents of children with severe intellectual disabilities in the UK, low parental awareness of respite services was associated with a lack of contact with social workers (Chadwick et al., 2002). Lower parenting confidence facilitated EI provision and receipt. For example, McConachie et al. (2001) found mothers with lower parenting confidence were more likely to access EI for their children with cerebral palsy in Bangladesh, compared to mothers with higher parenting confidence. Low parental readiness to take part when EI was offered was reported as a barrier by parents of children with ASD in New Zealand (Birkin et al., 2008). Language barriers made EI access difficult for families whose primary language differed from that of the primary language spoken in their area of residence, whereas increased language proficiency facilitated access in the USA (Bailey et al., 1999; Marshall et al., 2015). In Bailey et al. (1999), parental education modified language proficiency, as higher language proficiency was associated with increased educational level, but parent education did not directly influence EI receipt in their study.

Being a father, as opposed to a mother, was a barrier to EI receipt, modified by perceptions of parental roles, cultural norms, societal expectations, work schedules and timing (Evans et al., 2016; Herbert & Carpenter, 1994; Ridding & Williams, 2019). In a UK study, Herbert and Carpenter (1994) reported no support was directly offered to fathers, as EI was focused on support for the mother and child with Down syndrome. Similarly, a perceived disregard of fathers in terms of services provision (e.g. location, focus or timing of support), viewed as more mother‐orientated was identified as a barrier to accessing support by fathers of children with Down syndrome in the UK (Ridding & Williams, 2019). Mothers of children with developmental disabilities (developmental delay, cerebral palsy, behavioural needs, epilepsy) in Evans et al.’s study (2016) in the USA reported that fathers felt ‘uncomfortable’ interacting with professionals, deferring care to mothers and that fathers were more concerned with financially supporting the family and paying for EI, rather than participating in EI. Limited time and work schedules were specific barriers to fathers’ EI receipt (Evans et al., 2016; Herbert & Carpenter, 1994; Ridding & Williams, 2019).

Parental religion and faith also influenced EI receipt (Dababnah et al., 2019; Hussein et al., 2019). For example, some parents expressed a preference to access support from a religious healer rather than formal EI services, such as educational, medical or social services (Hussein et al., 2019; McConachie et al., 2001). In their small qualitative study of Somali parents in the UK, Hussein et al. (2019) reported a shift towards accessing both religious and formal EI, indicated in the following parent quote: ‘I think there has to be a balance, prayers are important but so is medical help’ (Hussein et al., 2019, p. 1414). Parents experiencing higher stress levels were more likely to access services, suggesting increased stress was a facilitator (Thomas et al., 2007). However, difficulties accessing services were reported as a unique source of stress, and so increased stress may also be a result of navigating complex systems to access EI (Mackintosh et al., 2012; McConachie et al., 2001).

The influence of the number of children in the household on EI provision or receipt varied modified by the type of EI and informal support available (Chadwick et al., 2002; Chauhan et al., 2017). For example, multi‐generational households enhanced access for some parents of children with developmental delays in India, as they received additional childcare support, but for others reduced their control over EI decisions (Chauhan et al., 2017). Parental time constraints were barriers to EI receipt, modified by family composition, employment status, work schedules, caring responsibilities, household duties and other time commitments (Evans et al., 2016; Marshall et al., 2015). In addition, due to the time required to navigate service systems and access EI (and difficulty or inability to access childcare), many parents reduced their working hours or left employment to care for their child, which reduced their economic resources (Marshall et al., 2015).

The nature and severity of need influenced EI receipt; greater severity of need generally facilitated EI provision, whereas lower severity was a barrier. For example, children with mild‐to‐moderate intellectual disabilities received less EI than children with profound‐to‐severe intellectual disabilities, whereas increased severity of behavioural needs acted as a facilitator or barrier to EI provision (Payakachat et al., 2017; Salomone et al., 2014). Bowker et al., 2011 found children with Asperger syndrome were less likely to access support compared to children with other ASD diagnostic labels. Whilst a trickle‐down effect of delayed recognition or identification of developmental disabilities may in part explain the relationship between severity of need and EI receipt, it appeared to be modified by other factors, such as service intake or eligibility criteria (Birkin et al., 2008; Twardzik et al., 2017).

Although older child age appeared to be a barrier to EI receipt, the type and amount of EI accessed varied dependent on child age. Whilst younger children (aged <3 years) with ASD accessed more hours of EI services, older children with ASD (aged 3–6 years) accessed more EI services overall (Payakachat et al., 2017). Female child gender was also a barrier to EI receipt for children with ASD (Payakachat et al., 2017), which may be attributed to barriers in the preceding steps of the pathway of access subsequently influencing EI receipt (e.g. delayed recognition or diagnosis).

#### 3.3.2 Services

Services factors that influenced EI receipt were the implementation of developmental surveillance, formal identification of need, referral practices, intake criteria and processes, professionals’ expertise, services capacity, funding, collaboration and coordination.

Similar to preceding phases, the implementation of developmental surveillance facilitated EI receipt for children with developmental delays (King et al., 2010). Formal identification of need (i.e. receipt of diagnosis or label from a professional) generally facilitated EI access (Chen et al., 2008; Jimenez et al., 2014; Payakachat et al., 2017). The diagnostic label given also influenced EI access, potentially related to the type or level of support needed. For example, in a Canadian study, children diagnosed with Asperger syndrome were less likely to access ASD supports compared to children diagnosed with other ASD diagnostic labels (Bowker et al., 2011).

Referral practices that facilitated EI provision included prompt follow‐up post‐diagnosis, sending referrals directly to services and actively supporting families to enrol in EI (e.g. contacting services, completing application forms) (Carr & Lord, 2016; Jimenez et al., 2014). Complex referral systems and placing responsibility on parents to contact services were barriers (Jimenez et al., 2014).

Strict eligibility criteria (services level or regionally) were a barrier, and broad eligibility criteria were a facilitator of EI access (Birkin et al., 2008; Twardzik et al., 2017). For example, Twardzik et al. (2017) found higher EI participation for children with developmental delays in US states with a broad eligibility policy compared to states with a narrow eligibility policy. Families reported being denied access to EI services if they did not meet specific eligibility criteria, such as if their child was ‘too young’ or ‘too old’ (Birkin et al., 2008; Mackintosh et al., 2012). Services intake procedures also influenced EI access. Low levels of EI access following the rollout of the NDIS in Australia was attributed to ‘broken down’ intake procedures before the NDIS became operational (Marchbank, 2017).

Increased professionals’ expertise (higher levels of training and training in developmental disabilities) facilitated EI provision, whereas lower expertise was a barrier (Brookman‐Frazee et al., 2012; Hudson et al., 2008). The provision of support to develop professionals’ expertise in developmental disabilities was a facilitator, such as providing mandatory and supplementary training, opportunities to work alongside experienced practitioners, and networking meetings (Hudson et al., 2008).

Limited capacity and availability of services and professionals were barriers to EI provision, including waiting lists and a lack of specialists (Birkin et al., 2008; Mackintosh et al., 2012). Funding barriers also obstructed EI provision, such as limited funding and resources within the system and unclear funding streams, modified by government legislation and budget allocation (Brookman‐Frazee et al., 2012; Marchbank, 2017; Ridding & Williams, 2019). For example, budget cuts increased barriers (Ridding & Williams, 2019), whereas legislation that stipulated EI funding reduced barriers (Brookman‐Frazee et al., 2012). In Mathews et al. (2018), the development of a robust funding and business model to sustain service provision facilitated EI receipt, in addition to identification. The provision of grants to enable services to fund EI professionals and families to cover EI‐related costs (e.g. travel, childcare) also facilitated EI entry in the USA (Hudson et al., 2008).

In addition to poor collaboration and communication between services and professionals, a lack of services coordination was also a barrier to EI receipt (Cassidy et al., 2008; Mathews et al., 2018). Establishing and maintaining partnerships and links between organisations facilitated EI provision, as it enabled collaboration and the opportunity to share resources (Carr & Lord, 2016).

#### 3.3.3 Intersection

Intersection factors that influenced EI receipt included the nature and flexibility of services delivery in relation to family factors, communication and contact between services and families, geographical accessibility and the intersection of EI content and family factors.

A good match between services delivery factors (services provided, costs, etc.) and family factors (nature of need, available resources, etc.) facilitated EI receipt, whereas a poor match was inevitably a barrier (Birkin et al., 2008; Chadwick et al., 2002). The ability of services to be flexible and adapt services delivery to meet the needs of families facilitated access. Flexibility and adaptability included providing services in accessible locations (e.g. local community centre, family home) or remotely (e.g. telephone, internet), delivering multiple services within a single location, providing EI in multiple delivery modes (e.g. reading, individual or group sessions), and offering services at suitable times (Carr & Lord, 2016; Hudson et al., 2008).

Communication barriers between professionals and families obstructed EI receipt, such as lack of information and guidance provided to families (Birkin et al., 2008; Howlin & Moore, 1997). Conversely, providing parents with practical information about local services and actively supporting contact with other services facilitated EI entry (Carr & Lord, 2016).

Similar to the identification phase, geographical proximity between families and services influenced EI provision in New Zealand (Birkin et al., 2008). A lack of services where families lived was a barrier to EI receipt in the UK, with access described as being subject to a ‘postal/zip code lottery’ (i.e. dependant on services available in the catchment area, rather than need) (Howlin & Moore, 1997; Ridding & Williams, 2019). Urbanicity appeared to influence the type of EI families accessed. For example, in Taiwan, Chen et al. (2008) found that families living in urban areas accessed more psychiatric services, whereas families living in non‐urban areas accessed services with specialities other than psychiatry.

A poor match between the content of EI programmes or support and family factors was a barrier, whereas a good match was a facilitator (Chauhan et al., 2017; Dababnah et al., 2019; McConachie et al., 2001). As previously described, parents generally sought services that aligned with their perceptions of developmental disabilities or need (Chauhan et al., 2017; Shyu et al., 2010). Consideration of the cultural and contextual background of families in the development of EI facilitated access for ‘hard‐to‐reach’ families, such as families including refugees or from ethnic minority groups (Dababnah et al., 2019). Modifications to increase the relevance of EI content for families also facilitated access. For example, modifications to a universal parenting programme, Incredible Years Parent Training, to increase its relevance for parents of children with developmental disabilities (e.g. additional content on functional assessment of behaviour problems) facilitated access (McIntyre, 2008). Further modifications to the Incredible Years Parent Training programme to increase the cultural relevance for Chinese parents (e.g. using the ‘growth mindset’ to encourage praise) also facilitated access (Kong & Au, 2018).

#### 3.3.4 Contextual

Political events, political unrest and government legislation influenced EI provision. For example, during an EI trial for Syrian refugees in Turkey, a terrorist attack and an attempted government coup (which increased animosity towards refugees) were barriers to EI receipt, as they reduced the security and safety of families in their community (Dababnah et al., 2019). Government legislation stipulating EI funding facilitated EI receipt, whereas the absence of government legislation requiring insurance companies to fund EI was reported as a barrier (Brookman‐Frazee et al., 2012).

## 4 DISCUSSION

In this paper, we described a framework depicting the pathway of access to EI across three key phases (recognition of potential need, identification or diagnosis, EI provision or receipt) and provided an overview of factors identified in the research literature found to influence EI access across this pathway for families of children with developmental disabilities. Whilst some factors operated similarly across the pathway, others appeared to operate differently across phases, and the impact a factor had was context dependent. For example, whilst older child age facilitated recognition and identification of ASD in children without intellectual disabilities, older child age was a barrier of EI receipt. The information brought together in our framework and literature review is a useful starting point to consider potential implications for policy, practice and future research, in relation to targeting investments to improve access to EI for families of children with developmental disabilities. Investments (financial or otherwise) to improve access to EI might have the most impact if targeted at factors which operate across multiple parts of the process. Conversely, factors which operate at only one phase might be useful for targeting individual or services level change.

Although we found that some factors influenced only one or two phases of the pathway of access to EI, it is likely that some influenced other phases but this evidence is simply not available due to gaps in the research evidence. For example, whilst the effect of staff turnover was only apparent at recognition, we found no studies in our narrative review that examined the influence of staff turnover on identification or EI provision. Similarly, although parental language proficiency (in relation to the prominent language spoken in their area of residence) emerged as influential to EI receipt, language barriers are likely to also influence recognition and identification. The influence of a factor at a specific phase may also trickle down to subsequent phases. Whilst this phenomenon was clearly demonstrated for developmental surveillance (the implementation of developmental surveillance facilitated recognition, which in turn increased the number of children with needs formally identified and receiving EI as a result), other factors may also influence the process of access to EI in similar ways. Further, although in our narrative review factors are primarily described individually in terms of their influence on the process of access to EI, it is very likely that the factors are not independent. Rather, several factors are likely to co‐occur and their influence on access is interrelated, such as the nature and severity of the need, ethnicity, culture, language and SES.

### 4.1 Implications

Our overview of factors influencing access to EI (Figure 2) can be used to inform the development of future research investigating rates of access to EI, in addition to barriers, facilitators, and moderators of access. Although we broadly discuss the implications of our findings below, access is context‐specific, and the complex relationship between various factors may vary accordingly. Our framework (Figure 2) can be applied to various contexts to develop a comprehensive understanding of access for a specific context. Several broad implications for policy and practice in relation to potential investments to increase access to EI emerged in this narrative review. In addition, our review suggests much needed research to explore gaps in the literature, develop this body of knowledge and explore ways to improve access to EI.

First, policies to reduce poverty (or the effect of poverty on access to services) have the potential to facilitate earlier recognition and identification of needs, in addition to EI receipt. Reducing poverty has also been identified as the top priority and greatest global challenge in the Sustainable Development Goals (United Nations, 2015). Whilst the specific steps required to reduce poverty or its impact on EI access will vary greatly across contexts (wealth of country, financial set‐up of service systems, etc.), policies could target poverty directly or focus on either subsidising service costs or providing universal free access to education, health, and social services. Universal free services provision has the potential to influence the entire EI access pathway, as increasing contact with services increases the opportunities for professionals to recognise potential needs, in addition to increasing families’ ability to access services to identify and meet needs. As families of children with developmental disabilities are more likely to experience poverty (Rosenberg et al., 2008), initiatives to remove or reduce economic barriers may be especially important to improve access to EI for this group. Our findings also highlighted the need to increase the capacity and availability of services that are universally free or heavily subsidised, and to ensure services are available in areas with high deprivation. Where this is not possible, such as in areas with limited or no public services, providing support with transport or remote access (e.g. telehealth), may reduce barriers.

As parents usually were the first to recognise potential delays or needs, investments to raise parental awareness of developmental disabilities and other family needs could be beneficial, paired with practical advice on what to do if they recognise a potential need. Future research should explore the time delay between initial parental concerns and seeking support, as further understanding can help identify strategies to reduce this. As parents with higher SES recognised needs earlier, interventions to increase awareness and knowledge could be most impactful if targeted at parents with lower SES. Although belonging to certain cultural and ethnic minority groups appeared to operate as a barrier to accessing formal EI, aspects of culture and ethnicity may act as protective factors for parental well‐being (Akbar & Woods, 2019). Due to the clear influence of cultural and contextual factors on perceptions of developmental disabilities and help‐seeking, factors identified in de Leeuw’s et al. (2020) review on ASD, which included non‐ASD literature, could be useful for designing research to understand access in non‐Western, low‐ and middle‐income countries. Key factors include cultural norms, beliefs and attitudes, mental health and child development literacy, goals of seeking clinical help and transference of information towards the clinician (de Leeuw et al., 2020). It would also be beneficial to raise awareness of developmental disabilities amongst various professionals who work with children and families. Investment to increase the skill and capacity of the workforce is vital to improve EI access and requires multifaceted approaches to recruitment, retention, and the provision of adequate training and support for professionals. Training should cover recognising various needs, screening methods, referral processes, diagnostic methods and EI supports (Kuriakose & Shalev, 2016), as well as communicating with and building partnership with families.

Increasing professionals’ awareness of children and families who are most at‐risk of delays to recognition and identification (e.g. low SES, ethnic minority groups, female gender) may also improve EI access (Kuriakose & Shalev, 2016). Exploration of research into the recognition or identification of developmental disabilities in middle‐late childhood or adulthood may also be important for understanding and addressing these barriers earlier (e.g. Huang et al., 2020). Our findings highlight the need for further research in differential symptom presentation, professionals’ knowledge of developmental disabilities and the sensitivity of screening tools. Developing guidelines and protocols for professionals to follow when parents raise a concern or if the professional suspect's developmental disabilities may facilitate progression from recognition to identification. Investment to roll out developmental surveillance and screening for all young children could improve access across recognition, identification and EI provision and receipt phases. Whilst this is particularly important for countries without universal screening systems, enhancing screening processes and methods is important for countries that implement universal screening. Universal screening may not be appropriate for different developmental disabilities. For example, there has been debate over the implementation of universal ASD screening, citing the potential benefits of early identification and EI, whilst raising concerns regarding the efficiency of screening tools (Mandell & Mandy, 2015). Therefore, a two‐pronged approach to surveillance is needed, implementing universal developmental screening in addition to improving the effectiveness and practicality of screening tools. It is crucial to provide training and support in the use of screening tools for professionals. To enable professionals to select the most appropriate methods, the suitability and effectiveness of screening (and diagnostic) tools at detecting developmental disabilities in different groups of children (e.g. gender, culture) and different contexts should be reviewed. A useful example is a recently published review of screening tools for developmental disabilities in the context of low‐ and middle‐income countries (Marlow et al., 2019).

Conducting surveillance and screening for other family needs (e.g. parenting support, parental stress, mental health, child behaviour problems, sibling adjustment) as part of routine monitoring may also be beneficial, especially for facilitating access to EI. This is key in a system designed to serve families, rather than just the child. Currently, EI mostly focuses on child needs, but as the family system influences child development and other EI outcomes (e.g. Totsika et al., 2019), the orientation of EI should focus on supporting the family system, as this is crucial for sustaining child outcomes (Brooks‐Gunn et al., 2000). Therefore, the success of EI should be defined by both child and family‐level outcomes (Bailey et al., 2008).

Assessment and diagnostic pathways need to be simplified with clear, transparent processes, referral practices and criteria. Services and families should be involved in the development of local services pathways, with incentives to increase collaboration and partnership in the process, and to agree the responsibilities of each service. Right from the start, professionals should clearly explain to families the reasons for assessment referral, what they can expect from the process, and the potential benefits of early identification and intervention. Reducing reliance on families to navigate the system is vital. Monitoring the implementation of assessment pathways is crucial, to ensure they are fit‐for‐purpose and followed by professionals.

Although there is a significant amount of research into families’ experiences across recognition to identification or diagnosis of developmental disabilities, especially ASD, there is a paucity of research that captures families’ experiences across all phases of the process. Whilst more comprehensive research is needed, following the identification of need, providing families with follow‐up sessions to ensure they have the opportunity to discuss needs with professionals and to provide them with useful information (e.g. about the need, benefits and goals of EI, how to access services, eligibility, financial supports), may be critical to improve access from identification to EI provision. Professionals/services should also provide families with practical support with access to EI (e.g. completing forms, emails, phone calls) and share up‐to‐date resources.

Investments to improve the intersection between services and family factors are also key to improving EI access. For example, providing multiple services in a single location with appointments coordinated across services could reduce the impact of several practical barriers of access (e.g. time, cost, travel). Employing culturally and linguistically diverse staff teams and ensuring EI content is culturally appropriate could reduce cultural barriers. The issue of limited services capacity in relation to need has to be addressed to increase the number of families served and reduce unacceptably long waiting times. Some actions to increase services capacity might be to: increase government funding for services, increase the size of the workforce with the skills to support families via training programmes and incentives, or to employ professionals to assess needs within existing services (e.g. specific to resource‐constrained settings, such as establishing small‐scale multidisciplinary teams and EI delivery by non‐specialists, see Divan et al., 2015, 2019; Khan et al., 2018).

There is a need to develop an understanding in local areas of families of children with developmental disabilities in terms of prevalence, geographical spread and demographics, to understand the match between services availability and local need. Considering the match between family factors, services provided (e.g. type of support, EI content) and services delivery factors (e.g. location, costs, time, delivery method) may be invaluable to increasing access. Subsequent plans to reduce barriers can be instigated, such as ensuring services are provided at accessible locations (including the family home or virtually, where appropriate), the types and intensity of supports match the varied needs of families, information about EI is accessible to different groups, and the content of EI is appropriate matched to needs. Tools are available to help services review and increase their accessibility for various families, such as the model of risk, disability and hard‐to‐reach families (Phoenix & Rosenbaum, 2019). It is vital that families are consulted with regard to the development or improvement of EI services.

Finally, investments to improve communication and partnership between services and families have great potential to facilitate access to EI. Communication barriers were prominent across the research evidence reviewed and had a detrimental impact on the process of access to EI, especially with regard to professionals’ responses to parental concerns. There is also emerging evidence that getting communication right is crucial to parents feeling that they are getting ‘good’ support (Stanford et al., 2020). As parents were usually the first to recognise developmental delays (and were generally right in their assessment, e.g., Bellman et al., 2013), ensuring professionals respond effectively to parental concerns is key to facilitating timely identification and access to EI. Increasing partnership between families and professionals may also be beneficial, especially in contexts with limited or reduced funding for services, such as the recent implementation of austerity by the UK government (Karim et al., 2012).

### 4.2 Limitations

Whilst our conceptual framework of access to EI (Figure 1) was simple so as to be inclusive of all service access within the early years, different factors influencing access may have been identified if a model of access to specific services was used, such as a framework of access specifically to healthcare services (Meade et al., 2015). We also acknowledge that our present paper may not reflect all available research evidence, as it was not a systematic review. However, it was intended as a starting point to conceptualise the process of access to EI and capture a diverse body of literature on factors that might influence this process for families of children with developmental disabilities.

Whilst conclusions drawn from the present review need to be considered within the context of the narrative review methodology, there is now room for empirical research and systematic reviews (with a clear pre‐registered protocol, systematic inclusion and exclusion criteria and quality assessment of the included literature) to rigorously investigate more focused questions on EI access for families of children with developmental disabilities.

## Supporting information

Table S1

## Footnotes

^1^ While autism spectrum disorder (ASD) is predominately used in the current paper in line with the Diagnostic and Statistical Manual of Mental Disorders–5, other autism diagnostic labels (Asperger syndrome, autism, Pervasive Developmental Disorder Not Otherwise Specified, Rett syndrome, autistic traits) are used to describe studies completed in the context prior to the widespread use of ASD as an all‐encompassing autism diagnosis in instances where autism diagnostic label is pertinent to the findings.
