# Supplementary material for: Factors influencing access to early intervention for families of children with developmental disabilities: A narrative review
Source: J Appl Res Intellect Disabil. 2020 Dec 22;34(3):695–711. doi: 10.1111/jar.12852 (PMC8246771; doi:10.1111/jar.12852)
Supplement: Supplementary file 1 — Table S1 [file JAR-34-695-s001.docx]

Supplement for Factors influencing access to early intervention for families of children with developmental disabilities: a narrative review

The present document is a supplement to the paper *Factors influencing access to early intervention for families of children with developmental disabilities: a narrative review*. Due to space limitations, only key references were included in in the manuscript. Table 1 below includes all references for factors identified in our review.

Table 1

*References for Factors that Affect the Pathway of Access to Early Intervention*

| Factor | References |
| --- | --- |
| *Family factors* | |
| Parental socioeconomic status | Bickel, Bridgemohan, Sideridis, & Huntington, 2015; Brett, Warnell, McConachie, & Parr, 2016; Chauhan, Prasad, Rai, & Khurana, 2017; Fountain, King, & Bearman, 2011; Howlin & Moore, 1997; Hudson, Cameron, & Matthews, 2008; Jimenez, Barg, Guevara, Gerdes, & Fiks, 2012; Jimenez et al., 2014; Keenan, Dillenburger, Doherty, Byrne, & Gallagher, 2010; Leininger & Levy 2015; Mackintosh, Goin-Kochel, & Myers, 2012; Mandell et al., 2009; Marshall, Tanner, Kozyr, & Kirby, 2015; McConachie et al., 2001; Moh & Magiati, 2012; Payakachat, Tilford, & Kuhlthau, 2017; Rosenberg, Zhang, & Robinson, 2008; Salomone et al., 2016; Thomas, Parish, Rose, & Kilany, 2012; Thomas, Zahorodny, et al., 2012; Vande Wydeven, Kwan, Hardan, & Bernstein, 2012 |
| Ethnicity and culture | Birkin, Anderson, Seymour, & Moore, 2008; Dababnah, Shaia, Campion, & Nichols, 2018; Evans, Feit, & Trent, 2016; Fountain et al., 2011; Hussein, Pellicano, & Crane, 2019; Jimenez et al., 2012; Magaña, Lopez, Aguinaga, & Morton, 2013; Mandell, Listerud, Levy, & Pinto-Martin, 2002; Mandell et al., 2009; Matheis & Matson, 2015; Moh & Magiati, 2012; Payakachat et al., 2017; Rosenberg et al., 2008; Rosenberg, Landa, Law, Stuart, & Law, 2011; Thomas, Zahorodny, et al., 2012 |
| Child age | Jimenez et al., 2012; King et al., 2010; Marshall et al., 2015; Matheis & Matson, 2015; Payakachat et al., 2017; Salomone et al., 2016 |
| Child gender | Begeer et al., 2013; Brett et al., 2016; Chen, Liu, Su, Huang, & Lin, 2008; Jimenez et al., 2014; Mandell et al., 2009; Mandy et al., 2012; Matheis & Matson, 2015; McConachie et al., 2001; Payakachat et al., 2017; Rynkiewicz et al., 2016; Shattuck et al., 2009; Siklos & Kerns, 2007 |
| Nature and severity of need | Adams et al. 2016; Bickel et al., 2015; Bowker, D’Angelo, Hicks, & Wells, 2011; Brett et al., 2016; Chadwick, Beecham, Piroth, Bernard, & Taylor, 2002; Crane, Chester, Goddard, Henry, & Hill, 2016; Howlin, Wing, & Gould, 1995; Jimenez et al., 2012, 2014; King et al., 2010; Maenner et al., 2016; Mandell, Novak, & Zubritsky, 2005; Mandell et al., 2009; Marshall et al., 2015; Matheis & Matson, 2015; Mayes & Calhoun, 2003; McGill, Papachristoforou, & Cooper, 2006; Moh & Magiati, 2012; Oswald, Haworth, Mackenzie, & Willis, 2017; Payakachat et al., 2017 Rosenberg et al., 2008 Rosenberg et al., 2011; Salomone et al., 2014, 2016; Shevell, Majnemer, Rosenbaum, & Abrahamowicz, 2001; Twardzik, Cotto-Negrón, & MacDonald, 2017; Zuckerman, Lindly, & Sinche, 2015 |
| Family history of developmental disabilities | Bickel et al., 2015; Matheis & Matson, 2015 |
| Child birth order | Bickel et al., 2015; Rosenberg et al., 2011 |
| Parental recognition and perceptions of need or EI | Bickel et al., 2015; Birkin et al., 2008; Chauhan et al., 2017; Evans et al., 2016; Hussein et al., 2019; Jimenez et al., 2012, 2014; McConachie et al., 2001; McIntyre, 2008b; Miller-Gairy & Mofya 2015; Salomone et al., 2014; Shyu, Tsai, & Tsai, 2010; Vande Wydeven et al., 2012 |
| Parental awareness of services | Birkin et al., 2008; Chadwick et al., 2002; Howlin &Moore, 1997; Jimenez et al., 2012, 2014; Keenan et al., 2010; Ludlow, Skelly, & Rohleder, 2012; Marshall et al., 2015; Vande Wydeven et al., 2012 |
| Family composition | Bickel et al., 2015; Chadwick et al., 2002; Chauhan et al., 2017; Jimenez et al., 2014 |
| Parental time | Chauhan et al., 2017; Evans et al., 2016; Jimenez et al., 2012; Ludlow et al., 2012; Marshall et al., 2015; McConachie et al., 2001; McIntyre, 2008a, 2008b; Montes & Halterman, 2008 |
| Parental awareness of developmental disabilities | Birkin et al., 2008; Hussein et al. 2019; Miller-Gairy & Mofya, 2015 |
| Parenting confidence | McConachie et al., 2001 |
| Parental readiness to take part in EI | Birkin et al., 2008 |
| Parents’ language | Bailey et al., 1999; Marshall et al., 2015 |
| Parental gender | Evans et al., 2016; Herbert & Carpenter, 1994; Kayfitz, Gragg, & Orr, 2010; Ly & Goldberg, 2014; Ridding & Williams, 2019 |
| Parental religion and faith | Dababnah, Habayeb, Bear, & Hussein, 2019; Hussein et al., 2019; Hussein et al., 2019; McConachie et al., 2001 |
| Parental stress | Mackintosh et al., 2012; McConachie et al., 2001; Thomas, Ellis, McLaurin, Daniels, & Morrissey, 2007 |
| *Services factors* | |
| Developmental surveillance | Dosreis, Weiner, Johnson, & Newschaffer, 2006; King et al., 2010; Nygren et al., 2012 |
| Services capacity and availability | Birkin et al., 2008; Dosreis et al., 2006; George, Kolodziej, Rendall, & Coiffait, 2014; Karim, Cook, & O'Reilly, 2012; King et al. 2010; Mackintosh et al., 2012; Marshall et al., 2015; Rosenberg et al., 2008; Roux et al., 2012; Sices, Feudtner, McLaughlin, Drotar, & Williams, 2003 |
| Funding | Brookman-Frazee, Baker-Ericzén, Stadnick, & Taylor, 2012; Brookman-Frazee, Drahota, Stadnick, & Palinkas, 2012; Hudson et al., 2008; Karim et al., 2012; King et al. 2010; Marchbank, 2017; Mathews et al., 2018; Ridding & Williams, 2019 |
| Professionals’ expertise | Brookman-Frazee, Baker-Ericzén, et al., 2012; Brookman-Frazee, Drahota, et al., 2012; Chen et al., 2008; Dosreis et al., 2006; Hudson et al., 2008; Kalkbrenner et al., 2011; Ludlow et al., 2012; Shevell et al., 2001; Vande Wydeven et al., 2012 |
| Screening methods and tools | King et al., 2010; Marshall et al., 2015; Roux et al., 2012; Sices, Stancin, Kirchner, & Bauchner, 2009 |
| Services collaboration and coordination | Cassidy, McConkey, Truesdale‐Kennedy, & Slevin, 2008; Carr & Lord, 2016; Jimenez et al., 2012; Mathews et al., 2018; Vande Wydeven et al., 2012 |
| Referral practices | Carr & Lord, 2016; Crane et al., 2016; Howlin & Moore, 1997; Jiminez et al., 2012, 2014; King et al., 2010; Marchbank, 2017; Oswald et al., 2017; Shevell et al., 2001; Vande Wydeven et al., 2012; Zuckerman et al., 2015 |
| Staff turnover | King et al., 2010 |
| Developmental surveillance processes | King et al., 2010; Nygren et al., 2012 |
| Identification methods and processes | Howlin & Moore, 1997; Jimenez et al., 2014; Karim et al., 2012; Moh & Magiati, 2012; Wiggins, Baio, & Rice, 2006 |
| Professionals’ recognition and perceptions of need | Begeer, El Bouk, Boussaid, Terwogt, & Koot, 2009; Burke, Koot, & Begeer, 2015; Burke, Koot, De Wilde, & Begeer, 2016; Crane et al., 2016; Cuccaro et al., 1996; Howlin & Moore, 1997; Karim et al., 2012; Oswald et al., 2017; Vande Wydeven et al., 2012; Zuckerman et al., 2015 |
| Formal identification of need | Bowker et al., 2011; Chen et al., 2008; Payakachat et al., 2017 |
| Eligibility criteria | Birkin et al., 2008; Mackintosh et al., 2012; Twardzik et al., 2017 |
| Service intake processes | Marchbank, 2017 |
| *Intersection factors* | |
| Nature and flexibility of service delivery | Birkin et al., 2008; Carr & Lord, 2016; Chadwick et al., 2002; Chauhan et al., 2017; Dababnah et al., 2019; Howlin & Moore, 1997; Hudson et al., 2008; Jimenez et al., 2012, 2014; Kong & Au, 2018; Mathews et al., 2018; McIntyre 2008a; Phaneuf & McIntyre, 2011; Roux et al., 2012 |
| Communication and contact | Birkin et al., 2008; Carr & Lord, 2016; Chadwick et al., 2002; Crane et al., 2016; Howlin & Moore, 1997; Jimenez et al., 2012, 2014; Keenan et al., 2010; Ludlow et al., 2012; Mackintosh et al., 2012; Marshall et al., 2015; Moh & Magiati, 2012; Oswald et al., 2017; Roux et al., 2012; Vande Wydeven et al., 2012; Zuckerman et al., 2015 |
| Geographic accessibility | Birkin et al., 2008; Chen et al., 2008; Howlin & Moore, 1997; Kalkbrenner et al., 2011; Keenan et al., 2010; Marshall et al., 2015; McConachie et al., 2001; Moh & Magiati, 2012; Ridding & Williams, 2019; Rosenberg et al., 2011; Salomone et al., 2016; Twardzik et al., 2017 |
| EI provision | Chauhan et al., 2017; Dababnah et al., 2019; Kong & Au, 2018; Magaña, Lopez, & Machalicek, 2017; McConachie et al., 2001; McIntyre, 2008a; 2008b |
| *Contextual factors* | |
| Political events/ unrest | Dababnah et al., 2019 |
| Government legislation | Brookman-Frazee, Baker-Ericzén, et al., 2012; Brookman-Frazee, Drahota, et al., 2012 |

References

Adams, D., Handley, L., Simkiss, D., Walls, E., Jones, A., Knapp, M., ... & Oliver, C. (2016). Service use and access in young children with an intellectual disability or global developmental delay: Associations with challenging behaviour. *Journal of Intellectual & Developmental Disability, 43*(2), 232-241.

Bailey, D. B., Skinner, D., Correa, V., Arcia, E., Reyes-Blanes, M. E., Rodriguez, P., ... & Skinner, M. (1999). Needs and supports reported by Latino families of young children with developmental disabilities. *American Journal on Mental Retardation, 104*(5), 437-451.

Begeer, S., El Bouk, S., Boussaid, W., Terwogt, M. M., & Koot, H. M. (2009). Underdiagnosis and referral bias of autism in ethnic minorities. *Journal of Autism and Developmental Disorders, 39*(1), 142-148.

Begeer, S., Mandell, D., Wijnker-Holmes, B., Venderbosch, S., Rem, D., Stekelenburg, F., & Koot, H. M. (2013). Sex differences in the timing of identification among children and adults with autism spectrum disorders. *Journal of Autism and Developmental Disorders,* 43(5), 1151-1156.

Bickel, J., Bridgemohan, C., Sideridis, G., & Huntington, N. (2015). Child and family characteristics associated with age of diagnosis of an autism spectrum disorder in a tertiary care setting. *Journal of Developmental & Behavioral Pediatrics, 36*(1), 1-7.

Birkin, C., Anderson, A., Seymour, F., & Moore, D. W. (2008). A parent‐focused early intervention program for autism: Who gets access? *Journal of Intellectual and Developmental Disability, 33*(2), 108-116.

Bowker, A., D’Angelo, N. M., Hicks, R., & Wells, K. (2011). Treatments for autism: Parental choices and perceptions of change. *Journal of Autism and Developmental Disorders, 41*(10), 1373-1382.

Brett, D., Warnell, F., McConachie, H., & Parr, J. R. (2016). Factors affecting age at ASD diagnosis in UK: no evidence that diagnosis age has decreased between 2004 and 2014*. Journal of Autism and Developmental Disorders, 46*(6), 1974-1984.

Brookman-Frazee, L., Baker-Ericzén, M., Stadnick, N., & Taylor, R. (2012). Parent perspectives on community mental health services for children with autism spectrum disorders. *Journal of Child and Family Studies, 21*(4), 533-544.

Brookman-Frazee, L., Drahota, A., Stadnick, N., & Palinkas, L. A. (2012). Therapist perspectives on community mental health services for children with autism spectrum disorders. *Administration and Policy in Mental Health and Mental Health Services Research, 39*(5), 365-373.

Burke, D. A., Koot, H. M., & Begeer, S. (2015). Seen but not heard: School-based professionals’ oversight of autism in children from ethnic minority groups. *Research in Autism Spectrum Disorders, 9*, 112-120.

Burke, D. A., Koot, H. M., De Wilde, A., & Begeer, S. (2016). Influence of child factors on health-care professionals’ recognition of common childhood mental-health problems. *Journal of Child and Family Studies, 25*(10), 3083-3096.

Carr, T., & Lord, C. (2016). A pilot study promoting participation of families with limited resources in early autism intervention. *Research in Autism Spectrum Disorders, 2*, 87-96.

Cassidy, A., McConkey, R., Truesdale‐Kennedy, M., & Slevin, E. (2008). Preschoolers with autism spectrum disorders: the impact on families and the supports available to them. *Early Child Development and Care, 178*(2), 115-128.

Chadwick, O., Beecham, J., Piroth, N., Bernard, S., & Taylor, E. (2002). Respite care for children with severe intellectual disability and their families: Who needs it? Who receives it? *Child and Adolescent Mental Health, 7*(2), 66-72.

Chauhan, S.,Prasad, P. L., Rai, P. L., & Khurana, B. (2017). Parental Perceptions Influencing the Utilization of Early Intervention Services in Children with Developmental Delay. *Journal of Nepal Paediatric Society 37*(1), 51-58.

Chen, C. Y., Liu, C. Y., Su, W. C., Huang, S. L., & Lin, K. M. (2008). Urbanicity-related variation in help-seeking and services utilization among preschool-age children with autism in Taiwan. *Journal of Autism and Developmental Disorders, 38*(3), 489-497.

Crane, L., Chester, J. W., Goddard, L., Henry, L. A., & Hill, E. (2016). Experiences of autism diagnosis: A survey of over 1000 parents in the United Kingdom. *Autism, 20*(2), 153-162.

Cuccaro, M. L., Wright, H. H., Rownd, C. V., Abramson, R. K., Waller, J., & Fender, D. (1996). Brief report: Professional perceptions of children with developmental difficulties: The influence of race and socioeconomic status. *Journal of Autism and Developmental Disorders, 26*(4), 461-469.

Dababnah, S., Habayeb, S., Bear, B. J., & Hussein, D. (2019). Feasibility of a trauma-informed parent–teacher cooperative training program for Syrian refugee children with autism. *Autism, 23*(5), 1300-1310.

Dababnah, S., Shaia, W. E., Campion, K., & Nichols, H. M. (2018). “We Had to Keep Pushing”: Caregivers' Perspectives on Autism Screening and Referral Practices of Black Children in Primary Care. *Intellectual and Developmental Disabilities, 56*(5), 321-336.

Dosreis, S., Weiner, C. L., Johnson, L., & Newschaffer, C. J. (2006). Autism spectrum disorder screening and management practices among general pediatric providers. *Journal of Developmental & Behavioral Pediatrics, 27*(2), 88-94.

Evans, D. L., Feit, M. D., & Trent, T. (2016). African American parents and attitudes about child disability and early intervention services. *Journal of Social Service Research, 42*(1), 96-112.

Fountain, C., King, M. D., & Bearman, P. S. (2011). Age of diagnosis for autism: individual and community factors across 10 birth cohorts. *Journal of Epidemiology & Community Health, 65*(6), 503-510.

George, C., Kolodziej, N., Rendall, M., & Coiffait, F. (2014). The effectiveness of a learning disability specific group parenting programme for parents of preschool and school-age children. *Educational & Child Psychology, 31*(4), 18-29.

Herbert, E., & Carpenter, B. (1994). Fathers‐the secondary partners: professional perceptions and fathers' reflections. *Children & Society, 8*(1), 31-41.

Howlin, P., & Moore, A. (1997). Diagnosis in autism: A survey of over 1200 patients in the UK. *Autism, 1*(2), 135-162.

Howlin, P., Wing, L., & Gould, J. (1995). The recognition of autism in children with Down syndrome‐implications for intervention and some speculations about pathology. *Developmental Medicine & Child Neurology, 37*(5), 406-414.

Hudson, A., Cameron, C., & Matthews, J. (2008). The wide-scale implementation of a support program for parents of children with an intellectual disability and difficult behaviour. *Journal of Intellectual & Developmental Disability, 33*(2), 117-126.

Hussein, A. M., Pellicano, E., & Crane, L. (2019). Understanding and awareness of autism among Somali parents living in the United Kingdom. *Autism, 23*(6), 1408-1418.

Jimenez, M. E., Barg, F. K., Guevara, J. P., Gerdes, M., & Fiks, A. G. (2012). Barriers to evaluation for early intervention services: parent and early intervention employee perspectives. *Academic Pediatrics, 12*(6), 551-557.

Jimenez, M. E., Fiks, A. G., Shah, L. R., Gerdes, M., Ni, A. Y., Pati, S., & Guevara, J. P. (2014). Factors associated with early intervention referral and evaluation: a mixed methods analysis. *Academic Pediatrics, 14*(3), 315-323.

Kalkbrenner, A. E., Daniels, J. L., Emch, M., Morrissey, J., Poole, C., & Chen, J. C. (2011). Geographic access to health services and diagnosis with an autism spectrum disorder. *Annals of Epidemiology, 21*(4), 304-310.

Karim, K., Cook, L., & O'Reilly, M. (2012). Diagnosing autistic spectrum disorder in the age of austerity. *Child: Care, Health and Development, 40*(1), 115-123.

Kayfitz, A. D., Gragg, M. N., & Orr, R. R. (2010). Positive experiences of mothers and fathers of children with autism. *Journal of Applied Research in Intellectual Disabilities, 23*(4), 337-343.

Keenan, M., Dillenburger, K., Doherty, A., Byrne, T., & Gallagher, S. (2010). The experiences of parents during diagnosis and forward planning for children with autism spectrum disorder. *Journal of Applied Research in Intellectual Disabilities, 23*(4), 390-397.

King, T. M., Tandon, S. D., Macias, M. M., Healy, J. A., Duncan, P. M., Swigonski, N. L., ... & Lipkin, P. H. (2010). Implementing developmental screening and referrals: lessons learned from a national project. *Pediatrics*, *125*(2), 350-360.

Kong, M. M. Y., & Au, T. K. F. (2018). The Incredible Years Parent Program for Chinese Preschoolers With Developmental Disabilities. *Early Education and Development, 29*(4), 494-514.

Leininger, L., & Levy, H. (2015). Child health and access to medical care. *Future Child, 25*(1), 65-90.

Ludlow, A., Skelly, C., & Rohleder, P. (2012). Challenges faced by parents of children diagnosed with autism spectrum disorder. *Journal of Health Psychology, 17*(5), 702-711.

Ly, A. R., & Goldberg, W. A. (2014). New measure for fathers of children with developmental challenges. *Journal of Intellectual Disability Research, 58*(5), 471-484.

Mackintosh, V. H., Goin-Kochel, R. P., & Myers, B. J. (2012). “What do you like/dislike about the treatments you’re currently using?” A qualitative study of parents of children with autism spectrum disorders. *Focus on Autism and Other Developmental Disabilities, 27*(1), 51-60.

Maenner, M. J., Schieve, L. A., Rice, C. E., Cunniff, C., Giarelli, E., Kirby, R. S., ... & Durkin, M. S. (2013). Frequency and pattern of documented diagnostic features and the age of autism identification. *Journal of the American Academy of Child & Adolescent Psychiatry, 52*(4), 401-413.

Magaña, S., Lopez, K., Aguinaga, A., & Morton, H. (2013). Access to diagnosis and treatment services among Latino children with autism spectrum disorders. *Intellectual and Developmental Disabilities, 51*(3), 141-153.

Magaña, S., Lopez, K., & Machalicek, W. (2017). Parents taking action: A psycho‐educational intervention for Latino parents of children with autism spectrum disorder. *Family Process, 56*(1), 59-74.

Mandell, D. S., Listerud, J., Levy, S. E., & Pinto-Martin, J. A. (2002). Race differences in the age at diagnosis among Medicaid-eligible children with autism. *Journal of the American Academy of Child & Adolescent Psychiatry, 41*(12), 1447-1453.

Mandell, D. S., Novak, M. M., & Zubritsky, C. D. (2005). Factors associated with age of diagnosis among children with autism spectrum disorders. *Pediatrics, 116*(6), 1480-1486.

Mandell, D. S., Wiggins, L. D., Carpenter, L. A., Daniels, J., DiGuiseppi, C., Durkin, M. S., ... & Shattuck, P. T. (2009). Racial/ethnic disparities in the identification of children with autism spectrum disorders. *American Journal of Public Health, 99*(3), 493-498.

Mandy, W., Chilvers, R., Chowdhury, U., Salter, G., Seigal, A., & Skuse, D. (2012). Sex differences in autism spectrum disorder: evidence from a large sample of children and adolescents. *Journal of Autism and Developmental Disorders, 42*(7), 1304-1313.

Marchbank, A. M. (2017). The National Disability Insurance Scheme: Administrators' perspectives of agency transition to 'user pay' for early intervention service delivery. *Australasian Journal of Early Childhood, 42*(3), 46-53.

Marshall, J., Tanner, J. P., Kozyr, Y. A., & Kirby, R. S. (2015). Services and supports for young children with Down syndrome: parent and provider perspectives. *Child: Care, Health and Development, 41*(3), 365-373.

Matheis, M., & Matson, J. L. (2015). Autism spectrum disorder screening refusal rates: Findings from a statewide early intervention program. *Journal of Developmental and Physical Disabilities, 27*(6), 755-770.

Mathews, T. L., Lugo, A. M., King, M. L., Needelman, L. L., McArdle, P. E., Romer, N., ... & Higgins, W. J. (2018). Expanding Access to Clinical Services for Toddlers with Autism Spectrum Disorders. *Journal of Pediatric Health Care, 32*(2), 173-183.

Mayes, S. D., & Calhoun, S. L. (2003). Ability profiles in children with autism: Influence of age and IQ. *Autism, 7*(1), 65-80.

McConachie, H., Huq, S., Munir, S., Akhter, N., Ferdous, S., & Khan, N. Z. (2001). Difficulties for mothers in using an early intervention service for children with cerebral palsy in Bangladesh. *Child: Care, Health and Development, 27*(1), 1-12.

McGill, P., Papachristoforou, E., & Cooper, V. (2006). Support for family carers of children and young people with developmental disabilities and challenging behaviour. *Child: Care, Health and Development, 32*(2), 159-165.

McIntyre, L. L. (2008a). Adapting Webster‐Stratton's incredible years parent training for children with developmental delay: findings from a treatment group only study. *Journal of Intellectual Disability Research, 52*(12), 1176-1192.

McIntyre, L. L. (2008b). Parent training for young children with developmental disabilities: Randomized controlled trial. *American Journal on Mental Retardation, 113*(5), 356-368.

Miller-Gairy, S., & Mofya, S. (2015). Elements of culture and tradition that shape the perceptions and expectations of Somali refugee mothers about autism. *International Journal of Child and Adolescent Health, 8*(3), 335-349.

Moh, T. A., & Magiati, I. (2012). Factors associated with parental stress and satisfaction during the process of diagnosis of children with autism spectrum disorders. *Research in Autism Spectrum Disorders, 6*(1), 293-303.

Montes, G. & Halterman, J. S. (2008). Child care problems and employment among families with preschool-aged children with autism in the United States. *Pediatrics, 122(*1), 202-208.

Nygren, G., Cederlund, M., Sandberg, E., Gillstedt, F., Arvidsson, T., Gillberg, I. C., ... & Gillberg, C. (2012). The prevalence of autism spectrum disorders in toddlers: a population study of 2-year-old Swedish children. *Journal of Autism and Developmental Disorders, 42*(7), 1491-1497.

Oswald, D. P., Haworth, S. M., Mackenzie, B. K., & Willis, J. H. (2017). Parental report of the diagnostic process and outcome: ASD compared with other developmental disabilities. *Focus on Autism and Other Developmental Disabilities, 32*(2), 152-160.

Payakachat, N., Tilford, J. M., & Kuhlthau, K. A. (2017). Parent-Reported Use of Interventions by Toddlers and Preschoolers With Autism Spectrum Disorder. *Psychiatric Services, 69*(2), 186-194.

Phaneuf, L., & McIntyre, L. L. (2011). The application of a three-tier model of intervention to parent training. *Journal of Positive Behavior Interventions, 13*(4), 198-207.

Ridding, A., & Williams, J. (2019). Being a dad to a child with Down’s syndrome: Overcoming the challenges to adjustment. *Journal of Applied Research in Intellectual Disabilities, 32*(3), 678–690.

Rosenberg, R. E., Landa, R., Law, J. K., Stuart, E. A., & Law, P. A. (2011). Factors affecting age at initial autism spectrum disorder diagnosis in a national survey. *Autism Research and Treatment, 2011,* 1-11.

Rosenberg, S. A., Zhang, D., & Robinson, C. C. (2008). Prevalence of developmental delays and participation in early intervention services for young children. *Pediatrics, 121*(6), 1503-1509.

Roux, A. M., Herrera, P., Wold, C. M., Dunkle, M. C., Glascoe, F. P., & Shattuck, P. T. (2012). Developmental and autism screening through 2-1-1: reaching underserved families. *American Journal of Preventive Medicine, 43*(6), 457-463.

Rynkiewicz, A., Schuller, B., Marchi, E., Piana, S., Camurri, A., Lassalle, A., & Baron-Cohen, S. (2016). An investigation of the ‘female camouflage effect’ in autism using a computerized ADOS-2 and a test of sex/gender differences. *Molecular Autism, 7*(1), 10.

Salomone, E., Beranová, Š., Bonnet-Brilhault, F., Briciet Lauritsen, M., Budisteanu, M., Buitelaar, J., ... & Fuentes, J. (2016). Use of early intervention for young children with autism spectrum disorder across Europe. *Autism, 20*(2), 233-249.

Salomone, E., Kutlu, B., Derbyshire, K., McCloy, C., Hastings, R. P., Howlin, P., & Charman, T. (2014). Emotional and behavioural problems in children and young people with autism spectrum disorder in specialist autism schools. *Research in Autism Spectrum Disorders, 8*(6), 661-668.

Shevell, M. I., Majnemer, A., Rosenbaum, P., & Abrahamowicz, M. (2001). Profile of referrals for early childhood developmental delay to ambulatory subspecialty clinics. *Journal of Child Neurology, 16*(9), 645-650.

Shattuck, P. T., Durkin, M., Maenner, M., Newschaffer, C., Mandell, D. S., Wiggins, L., ... & Baio, J. (2009). Timing of identification among children with an autism spectrum disorder: findings from a population-based surveillance study. *Journal of the American Academy of Child & Adolescent Psychiatry, 48*(5), 474-483.

Shyu, Y. I. L., Tsai, J. L., & Tsai, W. C. (2010). Explaining and selecting treatments for autism: Parental explanatory models in Taiwan. *Journal of Autism and Developmental Disorders, 40*(11), 1323-1331.

Sices, L., Feudtner, C., McLaughlin, J., Drotar, D., & Williams, M. (2003). How do primary care physicians identify young children with developmental delays? A national survey. *Journal of Developmental & Behavioral Pediatrics, 24*(6), 409-417.

Sices, L., Stancin, T., Kirchner, H. L., & Bauchner, H. (2009). PEDS and ASQ developmental screening tests may not identify the same children. *Pediatrics, 124*(4), 640-647.

Siklos, S., & Kerns, K. A. (2007). Assessing the diagnostic experiences of a small sample of parents of children with autism spectrum disorders. *Research in Developmental disabilities, 28*(1), 9-22.

Thomas, K. C., Ellis, A. R., McLaurin, C., Daniels, J., & Morrissey, J. P. (2007). Access to care for autism-related services. *Journal of Autism and Developmental Disorders, 37*(10), 1902-1912.

Thomas, P., Zahorodny, W., Peng, B., Kim, S., Jani, N., Halperin, W., & Brimacombe, M. (2012). The association of autism diagnosis with socioeconomic status. *Autism, 16*(2), 201-213.

Thomas, K. C., Parish, S. L., Rose, R. A., & Kilany, M. (2012). Access to care for children with autism in the context of state Medicaid reimbursement. *Maternal and Child Health Journal, 16*(8), 1636-1644.

Twardzik, E., Cotto-Negrón, C., & MacDonald, M. (2017). Factors related to early intervention Part C enrollment: A systematic review. *Disability and Health Journal, 10*(4), 467-474.

Vande Wydeven, K., Kwan, A., Hardan, A. Y., & Bernstein, J. A. (2012). Underutilization of genetics services for autism: the importance of parental awareness and provider recommendation. *Journal of Genetic Counseling, 21*(6), 803-813.

Wiggins, L. D., Baio, J., & Rice, C. (2006). Examination of the time between first evaluation and first autism spectrum diagnosis in a population-based sample. *Journal of Developmental and Behavioural Paediatrics, 27*(2), 79-87.

Zuckerman, K. E., Lindly, O. J., & Sinche, B. K. (2015). Parental concerns, provider response, and timeliness of autism spectrum disorder diagnosis. *The Journal of Pediatrics, 166*(6), 1431-1439.
